# The NIH Somatic Cell Genome Editing program

**DOI:** 10.1038/s41586-021-03191-1

**Published:** 2021-04-07

**Authors:** Krishanu Saha, Erik J. Sontheimer, P. J. Brooks, Melinda R. Dwinell, Charles A. Gersbach, David R. Liu, Stephen A. Murray, Shengdar Q. Tsai, Ross C. Wilson, Daniel G. Anderson, Aravind Asokan, Jillian F. Banfield, Krystof S. Bankiewicz, Gang Bao, Jeff W. M. Bulte, Nenad Bursac, Jarryd M. Campbell, Daniel F. Carlson, Elliot L. Chaikof, Zheng-Yi Chen, R. Holland Cheng, Karl J. Clark, David T. Curiel, James E. Dahlman, Benjamin E. Deverman, Mary E. Dickinson, Jennifer A. Doudna, Stephen C. Ekker, Marina E. Emborg, Guoping Feng, Benjamin S. Freedman, David M. Gamm, Guangping Gao, Ionita C. Ghiran, Peter M. Glazer, Shaoqin Gong, Jason D. Heaney, Jon D. Hennebold, John T. Hinson, Anastasia Khvorova, Samira Kiani, William R. Lagor, Kit S. Lam, Kam W. Leong, Jon E. Levine, Jennifer A. Lewis, Cathleen M. Lutz, Danith H. Ly, Samantha Maragh, Paul B. McCray, Todd C. McDevitt, Oleg Mirochnitchenko, Ryuji Morizane, Niren Murthy, Randall S. Prather, John A. Ronald, Subhojit Roy, Sushmita Roy, Venkata Sabbisetti, W. Mark Saltzman, Philip J. Santangelo, David J. Segal, Mary Shimoyama, Melissa C. Skala, Alice F. Tarantal, John C. Tilton, George A. Truskey, Moriel Vandsburger, Jonathan K. Watts, Kevin D. Wells, Scot A. Wolfe, Qiaobing Xu, Wen Xue, Guohua Yi, Jiangbing Zhou, The SCGE Consortium

**Affiliations:** 1 Department of Biomedical Engineering, University of Wisconsin-Madison, Madison, WI USA; 2 Department of Medical History & Bioethics, University of Wisconsin-Madison, Madison, WI USA; 3 Wisconsin Institute for Discovery, University of Wisconsin-Madison, Madison, WI USA; 4 McPherson Eye Research Institute, University of Wisconsin-Madison, Madison, WI USA; 5 RNA Therapeutics Institute, University of Massachusetts Medical School, Worcester, MA USA; 6 Office of Rare Diseases Research, National Center for Advancing Translational Sciences (NCATS), National Institutes of Health, Bethesda, MD USA; 7 Department of Physiology, Medical College of Wisconsin, Milwaukee, WI USA; 8 Department of Biomedical Engineering, Duke University, Durham, NC USA; 9 Merkin Institute of Transformative Technologies, Broad Institute of MIT and Harvard, Cambridge, MA USA; 10 Department of Chemistry and Chemical Biology, Harvard University, Cambridge, MA USA; 11 Howard Hughes Medical Institute, Cambridge, MA USA; 12 The Jackson Laboratory, Bar Harbor, ME USA; 13 Department of Hematology, St Jude Children’s Research Hospital, Memphis, TN USA; 14 Innovative Genomics Institute, University of California, Berkeley, Berkeley, CA USA; 15 Department of Chemical Engineering, Massachusetts Institute of Technology, Cambridge, MA USA; 16 Institute for Medical Engineering and Science, Massachusetts Institute of Technology, Cambridge, MA USA; 17 David H. Koch Institute for Integrative Cancer Research at the Massachusetts Institute of Technology, Cambridge, MA USA; 18 Department of Surgery, Duke University School of Medicine, Durham, NC USA; 19 Department of Earth and Planetary Sciences, University of California, Berkeley, Berkeley, CA USA; 20 Department of Neurological Surgery, Ohio State University, Columbus, OH USA; 21 Department of Bioengineering, Rice University, Houston, TX USA; 22 Russell H. Morgan Department of Radiology and Radiological Science, Division of MR Research, Johns Hopkins University School of Medicine, Baltimore, MD USA; 23 Cellular Imaging Section and Vascular Biology Program, Institute for Cell Engineering, Johns Hopkins University School of Medicine, Baltimore, MD USA; 24 Recombinetics, Inc, Eagan, MN USA; 25 Department of Surgery, Beth Israel Deaconess Medical Center, Boston, MA USA; 26 Department of Otolaryngology-Head and Neck Surgery, Harvard Medical School, Boston, MA USA; 27 Program in Neuroscience, Harvard Medical School, Boston, MA USA; 28 Eaton-Peabody Laboratory, Massachusetts Eye and Ear Infirmary, Boston, MA USA; 29 Department of Molecular and Cellular Biology, University of California, Davis, Davis, CA USA; 30 Department of Biochemistry and Molecular Biology, Mayo Clinic Rochester, Rochester, MN USA; 31 Department of Radiation Oncology, Washington University in St Louis, St Louis, MO USA; 32 Wallace H. Coulter Department of Biomedical Engineering, Georgia Institute of Technology, Atlanta, GA USA; 33 Stanley Center for Psychiatric Research, Broad Institute, Cambridge, MA USA; 34 Department of Molecular Physiology and Biophysics, Baylor College of Medicine, Houston, TX USA; 35 Department of Chemistry, University of California, Berkeley, Berkeley, CA USA; 36 Department of Molecular and Cell Biology, University of California, Berkeley, Berkeley, CA USA; 37 California Institute for Quantitative Biosciences (QB3), University of California, Berkeley, Berkeley, CA USA; 38 Howard Hughes Medical Institute, University of California, Berkeley, Berkeley, CA USA; 39 Molecular Biophysics and Integrated Bioimaging Division, Lawrence Berkeley National Laboratory, Berkeley, CA USA; 40 Gladstone Institute of Data Science and Biotechnology, Gladstone Institutes, San Francisco, CA USA; 41 Department of Medical Physics, University of Wisconsin-Madison, Madison, WI USA; 42 Wisconsin National Primate Research Center, University of Wisconsin-Madison, Madison, WI USA; 43 McGovern Institute for Brain Research, Massachusetts Institute of Technology, Cambridge, MA USA; 44 Division of Nephrology, University of Washington, Seattle, WA USA; 45 Kidney Research Institute, University of Washington, Seattle, WA USA; 46 Institute for Stem Cell and Regenerative Medicine, University of Washington, Seattle, WA USA; 47 Department of Medicine, University of Washington, Seattle, WA USA; 48 Department of Ophthalmology and Visual Sciences, University of Wisconsin-Madison, Madison, WI USA; 49 Horae Gene Therapy Center, University of Massachusetts Medical School, Worcester, MA USA; 50 Department of Medicine, Beth Israel Deaconess Medical Center, Boston, MA USA; 51 Department of Therapeutic Radiology, Yale University, New Haven, CT USA; 52 Department of Molecular and Human Genetics, Baylor College of Medicine, Houston, TX USA; 53 Division of Reproductive and Developmental Sciences, Oregon National Primate Research Center, Oregon Health and Science University, Beaverton, OR USA; 54 Pat and Jim Calhoun Cardiology Center, University of Connecticut School of Medicine, Farmington, CT USA; 55 Pittsburgh Liver Research Center, Department of Pathology, University of Pittsburgh School of Medicine, Pittsburgh, PA USA; 56 Department of Biochemistry and Molecular Medicine, University of California, Davis, Davis, CA USA; 57 Department of Biomedical Engineering, Columbia University, New York, NY USA; 58 Wyss Institute, Harvard University, Cambridge, MA USA; 59 Department of Chemistry, Carnegie-Mellon University, Pittsburgh, PA USA; 60 Biomarker and Genomic Sciences Group, National Institute of Standards and Technology, Gaithersburg, MD USA; 61 Department of Pediatrics, University of Iowa, Iowa City, IA USA; 62 Gladstone Institute of Cardiovascular Disease, Gladstone Institutes, San Francisco, CA USA; 63 Department of Bioengineering and Therapeutic Sciences, University of California, San Francisco, San Francisco, CA USA; 64 Office of Research Infrastructure Programs, Division of Program Coordination, Planning, and Strategic Initiatives, Office of the Director, National Institutes of Health, Bethesda, MD USA; 65 Department of Medicine, Massachusetts General Hospital, Harvard Medical School, Boston, MA USA; 66 Department of Bioengineering, University of California, Berkeley, Berkeley, CA USA; 67 Division of Animal Sciences, University of Missouri, Columbia, MO USA; 68 Robarts Research Institute and Department of Medical Biophysics, The University of Western Ontario, London, Ontario, Canada; 69 Department of Pathology, University of California, San Diego, La Jolla, CA USA; 70 Department of Biostatistics and Medical Informatics, University of Wisconsin-Madison, Madison, WI USA; 71 Department of Medicine, Brigham and Women’s Hospital, Boston, MA USA; 72 Department of Biomedical Engineering, Yale University, New Haven, CT USA; 73 Department of Biochemistry and Molecular Medicine, University of California, Davis, Davis, CA USA; 74 Department of Biomedical Engineering, Marquette University and Medical College of Wisconsin, Milwaukee, WI USA; 75 Morgridge Institute for Research, Madison, WI USA; 76 Department of Pediatrics, University of California, Davis, Davis, CA USA; 77 Department of Cell Biology and Human Anatomy, University of California, Davis, Davis, CA USA; 78 School of Medicine, University of California, Davis, Davis, CA USA; 79 California National Primate Research Center, University of California, Davis, Davis, CA USA; 80 Department of Nutrition, Case Western Reserve University, Cleveland, OH USA; 81 Department of Molecular, Cell and Cancer Biology, University of Massachusetts Medical School, Worcester, Worcester, MA USA; 82 Department of Biomedical Engineering, Tufts University, Medford, MA USA; 83 Department of Pulmonary Immunology, University of Texas Health Sciences Center at Tyler, Tyler, TX USA; 84 Department of Neurosurgery, Yale University, New Haven, CT USA

**Keywords:** Targeted gene repair, Genetics research

## Abstract

The move from reading to writing the human genome offers new opportunities to improve human health. The United States National Institutes of Health (NIH) Somatic Cell Genome Editing (SCGE) Consortium aims to accelerate the development of safer and more-effective methods to edit the genomes of disease-relevant somatic cells in patients, even in tissues that are difficult to reach. Here we discuss the consortium’s plans to develop and benchmark approaches to induce and measure genome modifications, and to define downstream functional consequences of genome editing within human cells. Central to this effort is a rigorous and innovative approach that requires validation of the technology through third-party testing in small and large animals. New genome editors, delivery technologies and methods for tracking edited cells in vivo, as well as newly developed animal models and human biological systems, will be assembled—along with validated datasets—into an SCGE Toolkit, which will be disseminated widely to the biomedical research community. We visualize this toolkit—and the knowledge generated by its applications—as a means to accelerate the clinical development of new therapies for a wide range of conditions.

## Abstract (web summary)

This Perspective discusses how the Somatic Cell Genome Editing Consortium aims to accelerate the implementation of safe and effective genome-editing therapies in the clinic.

## main

**Fig. 1:**
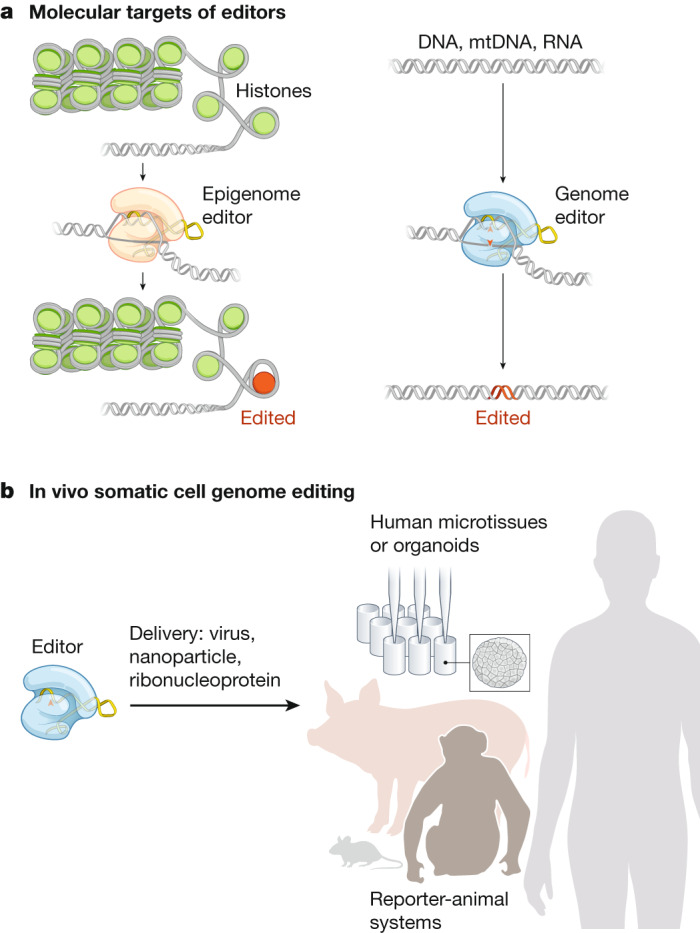
Tools for editing the genomes of cells within the body. Activities of the SCGE Consortium converge on editing the genome of cells inside the human body. **a**, Targets of the genome editors (right) range from DNA within the nucleus of a cell to other nucleic acids elsewhere within a cell, such as DNA within the mitochondria (mtDNA) or RNA in the cytoplasm. Targets of epigenomic editors (left) produce targeted alteration of the chromatin structure—including remodelling, modification of the 3D structure and the direct modification of histones or DNA—without editing the DNA or RNA sequence. Approaches to editing cells outside of the body, as well as germline editing in embryos, are not directly supported by the SCGE Consortium, nor are strategies for gene augmentation through the addition of exogenous DNA. **b**, Interoperable tools assembled into an SCGE Toolkit will be disseminated to accelerate the translation of safe and effective genome-editing therapeutics into the clinic. Tools encompass several categories: newly developed genome editors, delivery technologies, reporter-animal systems, and human biological systems.

Genetic factors contribute to most categories of human disease, including those that are inherited, infectious and malignant. It has therefore been a long-standing goal of biomedical science to develop a means to modify genomes within patients to correct disease-causing mutations, disable the genomes of invading pathogens, arm immune cells to attack tumours and enable countless other therapeutic opportunities. In some instances, gene addition can have therapeutic value, and gene therapy—the field that develops this approach—is experiencing ever-increasing success^1^. In many other cases, however, the genome of the patient must be edited to achieve therapeutic benefit. Genome editing broadly encompasses diverse technologies that can make many different genomic alterations in different contexts, and the topic has been the subject of recent and comprehensive reviews^2–4^. Several concepts in genome editing (Fig. 1) are central to the goals and strategies of the SCGE Consortium, which we describe in this Perspective.

Over the past few decades, a steady progression of techniques and technologies that enable user-programmable genome editing has been introduced, tested, improved and implemented. These include homologous recombination, zinc-finger nucleases (ZFNs), meganucleases and transcription activator-like effector nucleases (TALENs)^5–7^. Most recently, engineered molecular machinery^8–15^ derived from bacterial immune pathways—known as clustered regularly interspaced short palindromic repeats (CRISPR) and CRISPR-associated (Cas) proteins (CRISPR–Cas systems)^16^—have revolutionized genome editing^2–4^, in part because their target sequences can be simply programmed with easily designed RNA guides. Despite these promising advances, challenges remain before the transformative potential of therapeutic genome editing can be fully realized. Here we outline the goals and strategies of the SCGE Consortium, which has been established by the United States NIH to accelerate the development of solutions to many of these challenges. The NIH has allocated around US$190 million over 6 years in support of the SCGE Consortium, which now includes 72 principal investigators from 38 institutions that are pursuing 45 distinct but well-integrated projects.

Until the past decade, the most prominent genome-editing platforms (ZFNs, meganucleases, TALENs and Cas9/Cas12a systems) relied almost exclusively on the realization^17^ that the repair of nuclease-induced breaks in the genome can be exploited to induce genome edits (Fig. 1a)—either gene knockouts (through insertions or deletions generated by non-homologous end joining (NHEJ) or microhomology-mediated end-joining) or precise correction through homology-directed repair (HDR)^18^. Some editing events involve the insertion of vector-derived, ‘cargo’ sequences into the genome: natural examples of recombinases and transposases that can accomplish this task have been investigated for decades, and some (for example, lentiviral vectors) are being applied for gene therapy and genome editing^19^. In addition, some platforms can be implemented in partially or completely nuclease-inactive forms, by tethering to other effector proteins^20^. These strategies include base editing^21^ (in which fused deaminase enzymes rewrite individual nucleotides without inducing double-strand breaks)^22–24^ and prime editing (in which a fused reverse transcriptase introduces edits templated by an extended guide RNA)^25^. Nuclease-inactive forms can also be fused to enzymes to alter chromatin without changing the DNA sequence^26,27^ (Fig. 1a). Of course, no platform is appropriate for all contexts, and factors critical to editing success include efficiency (the fraction of the intended loci that are edited), precision (the relative frequency of desired (for example, reversion of a pathogenic allele) versus undesired (for example, large deletions or translocations) modifications at the intended loci) and accuracy (how many off-target sites are unintentionally edited, and to what extent).

Genome editing of somatic cells can be carried out either ex vivo, followed by the re-introduction of edited cells into the patient, or in vivo, by delivering the editing machinery to tissues within the body. An important distinction is the editing of somatic tissues versus germline tissues: the latter has the potential to transmit genetic changes to future generations. The SCGE Consortium is strictly focused on somatic editing; germline editing is not only excluded as a goal but is also considered to be an unacceptable outcome that should be carefully prevented.

## Existing capabilities and unmet needs

Genome-editing technologies have already demonstrated efficacy in diverse animal models of disease, including cancer, blood and metabolic disorders, inherited forms of blindness and deafness, and neuromuscular and neurological disease^2,28–30^. These successes have justified the move towards large animal models, in which signs of efficacy have also been found^31–33^. Early-stage clinical trials have shown that autologous edited cells can stably engraft and persist in humans^34–36^, and there have been early reports of the ex vivo editing of allogeneic T cells to fight cancer^37^ as well as the use of autologous haematopoietic stem cells to eliminate the need for blood transfusions in patients with sickle cell disease. However, ex vivo editing is logistically complex, expensive and hard to scale, given its requirement for substantial cell-manufacturing infrastructure. Therefore, in vivo approaches towards the editing of somatic cells are being developed^29^, and initial targets include cell types that are difficult to manufacture ex vivo (for example, in the retina (clinicaltrials.gov identifier NCT03872479) and in the liver (NCT03041324 and NCT04601051)). These studies highlight the great potential of genome editing to improve human health. However, they also underscore the need to address key limitations of these technologies. Specifically, in vivo editing still faces substantial hurdles in terms of efficacy and safety, especially in organ systems beyond the eye and the liver.

To achieve success in vivo, editors must be able to induce a range of edits to any target nucleic acid in the cell, including nuclear and mitochondrial DNA. Editors must be highly efficient but also safe, with acceptable levels of toxicity and minimal activation of innate immune responses. Adaptive immunity to either the editor^38–41^ or the delivery vehicle^42,43^ must also be managed, particularly in cases in which re-administration might be necessary to edit a desired proportion of a target cell population. Similarly, pre-existing immunity might need to be suppressed or circumvented in some cases^44–47^. A particularly daunting challenge is to develop delivery technologies that can ferry the editing machinery to numerous tissues in a safe and effective manner. We seek to better control the precise genomic changes that we intend to create at each targeted site, reduce the potential for unintended modifications at both targeted and non-targeted sites, and better understand the biological consequences of unintended editing events. These unmet needs are addressed by the initiatives of the SCGE Consortium, as elaborated below.

Despite the promise of changing any DNA sequence in the genome, the current programmable nucleases are most effective for gene knockout or for the excision of specific regions of genomic DNA. In fact, many gene-editing approaches for the treatment of diseases that are caused by mutations in a single gene—such as sickle cell disease, β thalassemia, Duchenne muscular dystrophy and Leber’s congenital amaurosis—are not intended to correct the inherited mutation or to restore the affected gene to a wild-type sequence. Instead, they are designed to knock out repressive genomic elements that will lead to the upregulation of compensatory factors^48^, to remove exons that will lead to the production of a partially functional gene product^49–51^, or to remove aberrant splice junctions^32^. The current inability to easily and accurately program specific sequences into the genome—given that HDR is largely ineffective in differentiated, post-mitotic cells^18^—is a fundamental obstacle to the broad use of genome editing in the treatment of genetic disease. Accordingly, new technologies that enable sequence-specific alterations—such as base editing^22,23^ and prime editing^25^—are also part of the SCGE Consortium’s portfolio of projects. In fact, base editing has already been used to correct pathogenic mutations, and in some cases has resulted in phenotypic rescue of the disease^52–63^.

Beyond new editing capabilities, there are numerous other technical limitations that must be overcome to advance the field. For example, there have been important advances in recent years in the prediction, characterization and validation of possible off-target editing^64^, building on foundational work with ZFNs^65^. Nonetheless, all of these methods are inherently incomplete, because it is not feasible to achieve non-destructive, whole-genome sequencing of every single edited cell. Similarly, most approaches are based on deep-sequencing technology, and are therefore limited by polymerase chain reaction biases, sensitivities, read lengths and the error rates of these methods. Moreover, off-target effects, unwanted events (for example, vector integrations, large deletions, rearrangements or translocations), genotoxicity and other adverse responses to genome editors might not be fully measurable in animal models. For these reasons, the development of methods to detect unwanted genomic events with increased predictive ability and sensitivity, as well as human cell and tissue systems such as organoids, are important components of the SCGE program.

The most substantial hurdle to the development of gene-editing therapies is the establishment of safe and effective delivery strategies. The genome-editing field can make use of four decades of innovation in the fields of gene therapy^1^ and nucleic acid therapeutics^66^, which have resulted in the development of numerous viral and non-viral delivery approaches. In fact, the recent regulatory approvals of gene therapies using both adeno-associated virus (AAV) and lentivirus vectors, as well as short interfering RNA (siRNA)-based and antisense-based drugs, provide lessons that are applicable to genome editors. However, many of the vectors that have been developed for gene therapy, which typically focuses on long-term expression to compensate for genetic defects, are not necessarily optimal for gene editing, which often requires transient delivery of editors. The most frequently used editors also introduce other challenges, including their large sizes (SpyCas9 and TALENs), their repetitive sequences and the need to deliver both components of a heterodimer (ZFNs and TALENs), and the requirement for delivery of a ribonucleoprotein complex (RNP; for example in CRISPR). Finally, the risk of on-target or off-target activity in inappropriate tissues underscores the need to ensure proper tissue targeting. Collectively, these challenges provide considerable opportunities for innovation.

## Goals of the SCGE Consortium

After reviewing the state of the field in 2017 through a series of stakeholder workshops, the NIH Common Fund noted needs that spanned multiple clinical indications, genes and target tissues^67^. The consensus was that the field needed new genome editors, delivery systems and biological systems to measure the safety and efficacy of various genome-editing strategies. The Common Fund subsequently launched the SCGE Consortium in 2018, by assembling a collection of multidisciplinary teams working on individual projects designed to address these needs.

The overarching goal of the SCGE Consortium is to accelerate the translation of genome-editing technology to a wide range of tissues and diseases. One of the key challenges in the field is the comparison of various technologies using common metrics and standards. For instance, a retinal delivery system might produce on-target indels at a gene of interest, but it is unclear whether the same delivery system could correct a different gene in the lung. Developmental paths that enable the mixing and matching of various technologies and readouts are woven into the SCGE program. In one example, all new delivery technologies developed in the first three years of the program will be tested first in small animals (for example, mice) and then—if successful—in large animals such as pigs and non-human primates. The resulting third-party data will be shared with the larger research community and with the public. A key value of the SCGE Consortium is transparency, which enables others to access its research output and use its results and products to inform and accelerate their own disease-focused projects. Along with data, we aim to deliver a collection of tools, reagents, methods and best-practices that will be assembled into the SCGE Toolkit for Therapeutic Genome Editing (or SCGE Toolkit in short, Fig. 1b). Through these activities and deliverables, the SCGE Consortium seeks to have a lasting impact by reducing the time and cost required to develop new therapies.

## Priorities and strategies

### Editing platforms

**Fig. 2:**
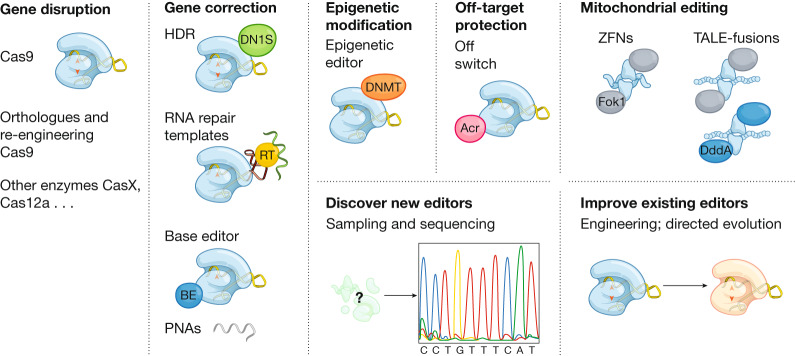
New genome editors in development. Major classes of genome editors include nucleases, base editors (BE), prime editors, PNAs, RNA editors and epigenome editors. The development of new editors involves mining metagenomic datasets and building upon existing editors, in part by tuning them for increased precision and accuracy. DNMT, DNA methyltransferase; Acr, anti-CRISPR protein; RT, reverse transcriptase; DN1S: dominant-negative mutant of tumour suppressor p53-binding protein 1, 53BP1; TALE-fusions, transcription activator-like effector-fusion with nucleases or cytidine deaminases (DddA).

Both the discovery of new gene-editing tools and their engineering continue to advance rapidly. As such, we seek to discover new editors and build upon existing editors, in part by tuning them for increased precision (Fig. 2). Although the bulk of SCGE studies will focus on the CRISPR system that is already in widest use (SpyCas9), as well as on other established Cas9 and Cas12a homologues^68–79^, it is imperative to continue to identify and test new systems and related tools. For example, new CRISPR–Cas systems to which humans have not previously been exposed^80^—as well as gene editors that are based solely on nucleic acid analogues that do not require protein cofactors^81^—could serve to circumvent detection by the immune system and also facilitate delivery. By searching through microbial data obtained from uncultivated samples, we hope to identify new systems that can be harnessed for the manipulation of DNA—such as helicases, nucleases, transposases, or recombinases^80,82–86^. These new systems could provide resources with improved efficiencies, alternative targeting mechanisms, smaller cargoes for viral packaging or decreased immunogenicity. This approach is exemplified by the recent development of Cas12j, the smallest CRISPR–Cas genome editing system yet discovered, which was supported by the SCGE program^87^.

In addition to the discovery of new CRISPR–Cas systems, we will continue to develop and improve engineered platforms—for example, base editing^21^—that efficiently edit genomes, including in post-mitotic cells and in mitochondrial DNA^24^. Well-established base editors can catalyse C-to-T transitions (cytosine base editors (CBEs))^22^, A-to-G transitions (adenine base editors (ABEs))^23^, or both^88–90^; very recently, C-to-G transversions in mammalian cells have also been enabled by base editing^91,92^. Ideally, programmed edits could change any nucleotide at any position in the genome; however, when using CRISPR–Cas effectors, editable bases are limited to regions that are near a compatible protospacer-adjacent motif sequence. Furthermore, editable sequences are restricted to a window that is a defined distance from the protospacer-adjacent motif. Through directed evolution, mining of natural variation or rational engineering, we aim to develop both broader targeting capabilities and increased specificity. Finally, we wish to eliminate limitations in changes to the targetable nucleotides. Prime editing, developed in part through the SCGE program, is an example of one such technology^25^.

Using CRISPR–Cas systems as ‘DNA cursors’ permits us to make edits not only to the DNA nucleotide sequence but also to the epigenetic marks that can alter gene-expression profiles and ultimately influence cellular function^93^. Like base editors, new CRISPR–Cas systems or variants that provide new binding sites can improve the accessibility of these new tools to all regions of the epigenome, and much has to be learned and developed to first understand and then to improve the specificity of epigenome editors. Such an approach extends the genome-engineering toolbox to apply to a much broader set of diseases, which can be addressed through changes in gene expression^93,94^ or through reprogramming cell phenotypes^95^. Epigenome-editing modalities have other potential advantages, including activating endogenous genes and networks for gain-of-function phenotypes, as well as tunability, reversibility and eliminating the possibility of off-target mutations or genotoxicity.

Although there is a considerable focus on CRISPR–Cas related systems within the SCGE Consortium, it is crucial to continue to explore alternative systems, in part because they could differ both in their potential for delivery and in the biological or immunological responses that they elicit. As one example, peptide nucleic acids (PNAs) are relatively small, synthetic molecules that recognize specific DNA sequences through triplex formation and subsequently induce editing^96^. The SCGE Consortium is developing improved methods for the production of PNAs, in addition to modifiers that could improve the function of PNAs for DNA editing, and a robust analysis of PNA function across many genetic loci^97,98^. Alternative systems could also target the many distinct mitochondrial genomes with human cells. These genomes are largely inaccessible to editing by systems that require guide RNAs or DNA donors, because of the current lack of reliable methods to transport these classes of molecules into mitochondria. The engineering of editors that target mitochondrial DNA could open up genome-editing therapies for the treatment of mitochondrial diseases, which affect 1 in approximately 5,000 people^24,99^. The discovery of DddA—an interbacterial toxin that catalyses the unprecedented deamination of cytidines within double-stranded DNA—led to the development of RNA-free DddA-derived CBEs (DdCBEs), which enabled the first purposeful sequence changes in mitochondrial DNA^24^. In addition to DdCBEs, other protein-based tools such as zinc fingers^100–102^ and TAL-like effectors^103,104^ are being fused to nucleases to control mitochondrial genome heteroplasmy.

### Delivery systems

Regardless of the genome-editing system that is selected to edit a particular therapeutic locus, its translation to the clinic is currently limited by the capacity for the editing payload to reach the nuclei of target cells. This translational bottleneck presents multifaceted challenges that differ from one target tissue to the next. An ideal delivery platform would be capable of conveying the required macromolecular components across cellular boundaries and into the nucleus; able to induce therapeutically useful levels of editing; amenable to cost-efficient, reproducible and scalable production; specific for particular cell types; and consistent with acceptable thresholds of toxicity, genotoxicity and immunogenicity. Failure to satisfy any of these criteria could render candidate delivery strategies ineffective, inaccessible or unsafe. After decades of research effort dedicated to the therapeutic delivery of DNA or RNA, viral vectors and lipid nanoparticles have emerged as promising platforms^105–107^ through which to deliver genome-editing machinery. However, many existing platforms have practical limitations for clinical use, as highlighted by the modest supply of genetic therapies in spite of extensive academic and industrial efforts. For example, the clinical use of AAV as a vector for the delivery of DNA that encodes the components of an editor (for example, a Cas protein effector and its guide RNA) is hampered by manufacturing bottlenecks, limited target-tissue tropisms, insertional mutagenesis and the immunogenicity of viral proteins^106^. For CRISPR systems in particular, the restricted genome-packaging capacity can be another major issue^106^. Nanoparticles that consist of cationic and hydrophobic molecules, loaded with messenger RNA (mRNA) and guide RNA cargo, provide alternative strategies and can be just as effective as viral vectors in terms of editing efficiency^108–110^. However, the broad application of genome editing will require nanoparticles that can target the many different types of tissue in the body.

To address these needs, the SCGE Consortium is working on 20 distinct projects that will explore new methods for the delivery of genome-editing machinery to specific tissue types in vivo (Table 1). Existing viral vectors are being enhanced with improved tissue-targeting capacity, enabling high efficacy at lower doses. Similarly, nanoparticles are being augmented with molecules that drive cell-type-specific association, generating powerful homing systems that can be administered intravenously or locally. The delivery of pre-formed CRISPR RNPs has shown the capacity for editing of respiratory epithelial cells using amphiphilic cell-penetrating peptides^111^, retinal cells^112^ and neurons in the brain^113^, for which convection-enhanced delivery might augment tissue distribution. A hybrid approach will pair nanoparticles with an AAV that carries template DNA to facilitate HDR^114^. Virus-like particles constitute a chimeric strategy: virally derived carriers are packaged with pre-formed RNPs, potentially maintaining delivery efficiency without the prolonged expression of editing machinery that is potentially associated with increased genotoxicity and immunogenicity. Other promising strategies include the use of extracellular vesicles, ultrasound, amphiphilic cell-penetrating peptides or chemical modifications of RNA components^105,107,115^ to improve targeted in vivo delivery (Table 1).

**Table 1:** Delivery systems under development

Delivery system	Target tissue	Administration	Cargo class^a^	PI(s)^b^
Viral: AAV	Brain	Intravenous	DNA	B. E. Deverman
Viral: AAV	Endothelium	Intravenous	DNA	G. Bao, W. R. Lagor
Viral: adenovirus	Endothelium	Intravenous	DNA	D. T. Curiel
Viral: AAV	Brain, skeletal muscle	Intravenous	DNA	A. Asokan, C. Gersbach
Non-viral: engineered guide RNAs	Brain	Local injection	RNP	E. J. Sontheimer, A. Khvorova, J. K. Watts, S. A. Wolfe
Non-viral: polymeric NP	Bone marrow, lung	Intravenous	mRNA, PNA	W. M. Saltzman, P. M. Glazer
Non-viral: polymeric NP	Brain	Local injection, intravenous	RNP	S. Gong, M. Emborg, J. E. Levine, S. Roy, K. Saha
Non-viral: polymeric NP	Brain	CED, intravenous	RNP	J. Zhou
Non-viral: cell-targeted NP	HSPCs	Intravenous	mRNA	J. Dahlman, P. J. Santangelo
Non-viral: liposomal NP	Inner ear	Local injection	mRNA, RNP	Z. Chen, D. R. Liu, Q. Xu
Non-viral: extracellular vesicles	Bone marrow	Intravenous	mRNA, RNP	I. Ghiran
Non-viral: PEGylated particles	Brain	CED	RNP	K. S. Bankiewicz, N. Murthy
Non-viral: ultrasound	Brain	Intravenous	DNA, RNP	K. W. Leong
Non-viral: amphiphilic peptides	Lung epithelium	Nasal instillation	RNP	P. McCray
Non-viral: engineered RNP	Immune cells	Intravenous	RNP	R. Wilson, J. A. Doudna
Non-viral: engineered RNP, VLP	HSPCs	Intravenous	RNP	E. Chaikof
Non-viral: engineered capsids	Intestinal cell types	Oral, intravenous	DNA, mRNA, RNP	K. Lam, R. H. Cheng
Non-viral: VLP	T cells	Intravenous	RNP	G. Yi
Non-viral: VLP	Lung, gastrointestinal tract	Intravenous	RNP	J. C. Tilton, M. Drumm, C. Flask, Z. Wang
Hybrid: NP and AAV	Lung epithelium	Inhalation/intratracheal	DNA, mRNA	G. Gao, D. G. Anderson, W. Xue

CED, convection-enhanced delivery; HSPCs, haematopoietic stem and progenitor cells; NP, nanoparticle; VLP, virus-like particle.

^a^‘Cargo’ refers to the molecular form(s) of genome-editing enzyme component(s): DNA encoding protein and guide RNA, mRNA encoding protein co-delivered with guide RNA, a RNP complex or a PNA.

^b^The lead principal investigator (PI) of the project is listed first. Additional PIs follow, listed alphabetically by last name.

### Testing in animals

Animal models provide essential validation of delivery systems within a living organism. Such models also serve as a proving ground for new therapeutics and a detection system for adverse events, including toxicity and immunogenicity. Target-indication-specific in-animal efficacy and safety studies are currently treated as essential by regulatory authorities in the United States and the European Union for nearly all genome-editing therapeutics that are being advanced to the clinic. One goal of the SCGE program is to generate in vivo reporter systems that are broadly applicable to many delivery systems and editing technologies, independent of the target cell or tissue type, or the specific disease to be corrected. These reporters should have the ability to detect and quantify genome editing in the intended target tissue, as well as editing events that result from non-specific delivery to other tissues throughout the body.

Small- and large-animal testing centres (SATCs and LATCs, respectively) within the SCGE Consortium centralize expertise with animal models (Table 2) to aid investigators in assessing the efficiency, specificity and safety of new delivery formulations in both wild-type and reporter-animal models. For example, the two SATCs are developing mouse reporter systems because mice are an ideal tool for the preliminary testing of new delivery formulations given their small size, low costs and well-established utility. Large animals are required for preclinical determination of safety, efficiency, dosing and reagent distribution, and as alternatives to mouse models when mice do not adequately recapitulate human responses. Engineered nucleases have enabled efficient and accurate genetic modification of large animals, such as non-human primates and pigs. Three research teams in the SCGE Consortium are developing large animal in vivo reporter systems: one group is dedicated to pigs and two others are dedicated to non-human primates, specifically marmosets and rhesus monkeys. The role of the LATCs is to assess the efficiency and safety of in vivo genome editing and delivery technologies, initially in wild-type animals. When the research teams that create and evaluate the reporter animals have accomplished their goals, they will provide the reporter animals to the LATCs to conduct independent validation and to establish large cohorts for the testing of genome editors.

**Table 2:** Animal testing systems under development

Organism	Editing events detected	Primary readout	Secondary readout	Editors	PIs^a^
Mouse	NHEJ, HDR, off-target cutting	Fluorescent signal in situ	Luciferase	SpyCas9, SauCas9, Cas12a	J. D. Heaney, M. E. Dickinson, W. R. Lagor
Mouse	NHEJ, HDR, base editing, PNA	Fluorescent signal in situ	Luciferase, NaI symporter	SpyCas9, SauCas9, Cas12a, Nme2Cas9, CjeCas9, ABE, CBE, PNA	S. A. Murray, C. M. Lutz
Pig	NHEJ, HDR	Fluorescent signal	NaI symporter	SpyCas9, SauCas9, Cas12a, ABE	D. F. Carlson; K. D. Wells, R.S. Prather
Macaque	NHEJ, HDR, C base editing	Fluorescent signal	Luciferase	SpyCas9, SauCas9, Cas12a, CBE	J. D. Hennebold; A. F. Tarantal, D. J. Segal
Marmoset	NHEJ	Akaluciferase	Fluorescence	SpyCas9, SauCas9, Nme2Cas9, Cas12a, ABE	G. Feng; A. F. Tarantal, D. J. Segal

NaI, sodium iodide.

^a^The lead PI of the project is listed first. Additional PIs follow, listed alphabetically by last name. Reporter Development and Testing Center teams are separated by semicolons.

The reporter-animal models are designed to faithfully activate in all cells and tissues in response to a specific gene-editing event. Fluorescent proteins provide a simple and robust means to detect activity at the single-cell level in situ, enabling identification of the specific cell types that are targeted. Reporters can be designed to detect different types of editing activity, often with a multi-functional arrangement to enable user flexibility. This includes nuclease activity through the detection of NHEJ-mediated repair events, as well as HDR of an inactivated reporter protein. The capacity to detect the activity of multiple nucleases (for example, SpyCas9, Cas12a and others) is highly desirable to enable comparative studies. Embodying these principles, SCGE reporter systems (Table 2) are primarily designed as improved variations of the Ai9 system^50,116^ or have a ‘traffic-light reporter’ design^117,118^. Other models will detect the activities of other types of editors, including ABEs and CBEs^21^ and PNA-based editing systems^96–98^. Additional reporter cassettes, such as Akaluciferase^119^ or sodium iodide symporters^120^, will be included to permit longitudinal detection by distinct imaging platforms. Importantly, all new reporter animals created as part of the SCGE program will be available for distribution to the wider biomedical community.

Along with the development of new model organisms, new non-invasive methods are needed to measure editing-associated outcomes. The SCGE Consortium is developing techniques for in vivo cell tracking using advanced imaging methods, including total-body positron emission tomography (PET) imaging^121^, magnetic particle imaging (MPI)^122^ and chemical exchange saturation transfer magnetic resonance imaging (CEST MRI)^123^, as outlined in Table 3. Ongoing projects will enable quantitative tracking of the locations and the fates of genome-edited cells after in vivo implantation^124–127^ or administration using cell labelling (MPI/MRI)^128^ and reporter gene (MRI/PET)^129–132^ approaches. Additionally, tracking the delivery and transduction of gene-editing cargo using AAV capsid proteins as endogenous CEST MRI contrast mechanisms is being examined. Each of these methods can be performed alongside existing, standard, non-invasive imaging to assess maladaptive responses to treatment. Together, these tools will provide a powerful combination of methods to quantify and link the delivery of gene-editing technology or gene-edited cells with subsequent biological outcomes.

**Table 3:** In vivo cell monitoring and in vitro human biological systems under development

In vivo cell monitoring		
Cell and tissue target	Reporter and/or contrast mechanism	PI(s)
hiPS cells in CNS	Tri-modal: iron oxide nanoparticle labelling and tracking for MRI + MPI and ^18^F-DCFPyL for PET	J. W. M. Bulte
Cardiac and hepatic tissues	AAV2 capsid as an endogenous contrast agent Genetically encoded reporter: lysine-rich protein	M. Vandsburger
CAR-T cells	Genetically encoded reporter genes MRI: OATP1B3 PET: NaI symporter	J. A. Ronald
Whole body, muscle and liver	Genetically encoded reporter: HSV-sr39tk Probe: ^18^F-FHBG	A. F. Tarantal, D. J. Segal
**Human biological systems**		
**Tissue**	**Cell source**	**PI(s)**
Brain	WTC11 hiPS cells	T. C. McDevitt
Heart	WTC11 hiPS cells	J.T. Hinson; T.C. McDevitt
Liver	WA09 hES cells, WTC11 hiPS cells	S. Kiani; T. C. McDevitt
Haematopoietic	Primary T cells	S. Q. Tsai
Eye	WA09 hES cells	K. Saha, D. M. Gamm, S. Roy, M. C. Skala
Muscle	hiPS cells, primary myoblasts, primary immune cells	C. A. Gersbach, N. Bursac, G. A. Truskey
Kidney	WTC11 hiPS cells, BJFF hiPS cells, WA09 hES cells	B. S. Freedman; R. Morizane, J. A. Lewis, V. Sabbisetti

CNS, central nervous system; ^18^F-DCFPyL, 2-(3-{1-carboxy-5-[(6-[^18^F]fluoro-pyridine-3-carbonyl)-amino]-pentyl}-ureido)-pentanedioic acid; ^18^F-FHBG, 9-(4-[^18^F]fluoro-3-hydroxymethylbutyl)guanine substrate for mutant herpes simplex virus type 1 thymidine kinase (HSV1-sr39TK); OATP1B3, human organic anion transporter polypeptide 1B3; CAR, chimeric antigen receptor; hES cells, human embryonic stem cells; hiPS cells, human induced pluripotent stem cells.

^a^The lead PI is listed first. Additional PIs follow, listed alphabetically by last name. Teams are separated by semicolons.

### Testing in human biological systems

The development of human biological systems to detect and minimize unintended biological effects of genome editing is a major focus of the SCGE Consortium. Although substantial progress has been made regarding methods for defining the genome-wide off-target mutations induced by genome editors^65,133–140^, as well as unintended outcomes (such as large deletions and rearrangements) at the on-target site, the interpretation of potential biological consequences associated with these mutations within human cells remains a major challenge^141^. Additionally, other effects of the editors or of the delivery components themselves—including the potential to stimulate immune responses^38,39,43–45,142^—have not been fully characterized. The SCGE Consortium is working to develop human cell-based and organoid platforms to define the unintended biological effects of editing (Table 3).

Projects use human primary cells when possible. For example, a primary T cell platform will define some of the unintended biological effects of genome editors. T cells are readily amenable to sensitive, unbiased methods for defining the genome-wide activities of editors^143^, and unique genomic rearrangements that establish a diverse T cell receptor repertoire can serve as cellular barcodes, facilitating single-cell analysis. Ex vivo screens for a T cell adaptive immune response to editors can also be readily implemented. When primary cells from the relevant target tissue are limiting, bioengineers within the SCGE Consortium will use self-renewing human cells to construct functional three-dimensional organoids or microphysiological systems. These platforms can bring together multiple cell types and extracellular matrices in a tissue-like architecture, providing an in vitro mimic of complex human tissues. Relevant functional assays with these systems can be defined, such as force generation from skeletal muscle, contraction of cardiac tissue and phototransduction in retinal organoids^144,145^. These systems can be scaled up to enable studies at higher throughput than would be feasible in animal models, and can also facilitate deeper molecular characterization of the various outcomes after editing different human cell types within a tissue. An ultimate aim of these studies is to produce assays that are relevant to regulatory science, to better evaluate various genome-editing strategies. Previous studies involving immunodeficient mice and edited T cells have been broadly enabling for many immune-cell-therapy products, and any new biological system that is developed by the SCGE Consortium, once established, could be similarly enabling for studies aiming towards investigational new drug filings in that cell and gene-therapy space.

### Integration of SCGE technologies

The initiatives described above—new editing platforms, delivery technologies, in vivo reporter systems and human biological systems—are expected to recombine and synergize in multiple ways, both planned and spontaneous. One prominent example, arising from the ever-growing recognition of the need for greater reproducibility during clinical translation^146^, is an explicit requirement for independent validation for all of the delivery technologies in development. Each delivery project involves multiple phases: an initial phase that establishes proof-of-principle within each laboratory that is developing a delivery technology; an intermediate phase that involves testing at a SATC, performed by SATC personnel; and finally, for those technologies that meet pre-defined SATC efficacy milestones, scale-up of the delivery system and testing at a LATC. Large-animal testing is contingent upon successful independent validation of the technology by an SATC—that is, outside of the laboratory that developed the delivery system in question. In another example, investigators that are developing new editors might choose to apply newly developed delivery systems to enable testing in vivo; delivery groups might use human biological systems to assess performance and adverse consequences before commencing with animal tests; and human biological systems could be developed to assess the editing precision of new editing platforms. Such cross-team, integrated projects are nurtured through internal calls within the SCGE Consortium to discuss collaborative proposals each year.

### Standards and the SCGE Toolkit

Although the above activities will generate a wide array of data and tools, the maximal impact will be achieved only when SCGE technologies use common standards and are interoperable. Data and resource standards and shared lexicons are imperative for the development of new technologies, and will be particularly critical for translating genome-editing systems into approved therapies^147,148^. To ensure the highest-quality data, interoperability of tools and reproducibility, the SCGE Consortium’s Dissemination and Coordinating Center (DCC) serves as a communication hub, facilitates collaboration among consortium members and builds platforms to enable the sharing of SCGE program resources and data, including through the SCGE Toolkit. Furthermore, to contribute to standards development, the SCGE Consortium is interfacing with the Food and Drug Administration, the National Institute of Standards and Technology (NIST) and the Defence Advanced Research Projects Agency. In particular, the SCGE Consortium is a member of the NIST Genome Editing Consortium.

The SCGE Toolkit (Fig. 1b) will be generated to develop the infrastructure and data to promote collaborations among the different projects within the SCGE Consortium, and to create a platform for investigators (and eventually, the broader scientific community and the public) to access data generated by the program. To ensure data integration and functional mining tools, standardized data formats and vocabularies are being developed and will be made available through the SCGE Consortium website. There will be several components of the SCGE Toolkit, including a public Resource Portal to provide both consortium members and other investigators with a single stop for information on existing data repositories, public tools and algorithms used in genome-editing research. Investigators within the SCGE Consortium will submit data to these existing resources when available. As these components are tested, validated and used together in experimental procedures, they will be integrated into a centralized database for both the SCGE Consortium and the public, facilitating the comparison of results across experiments and enabling researchers to further refine experimental designs for genome-editing research. Because much of the ongoing clinical development of genome editing is occurring within industry, the SCGE Consortium seeks to contribute broadly accessible data, tools, systems and assays that could enable a more open-access approach for clinical development.

## Outlook

New opportunities for the clinical translation of genome-editing technologies are arising from a deeper understanding of the human genome and from rapidly advancing bioengineering capabilities. The SCGE Consortium aims to develop new technologies and adapt existing tools to take immediate advantage of these opportunities, define and mitigate safety risks, and extend therapeutic genome editing into the most challenging somatic tissue contexts. Previous large-scale projects^149–155^ advanced the frontiers of genomics not only by producing new knowledge, but also by developing a common framework that ensured reproducibility, applied common standards and established the interoperability of distinct technologies. Inspired by these efforts, the SCGE program is designed to advance the field of genome editing towards a broadened spectrum of human therapeutic applications.

## Supplementary information

Supplementary Information Grouped membership list of the five SCGE initiatives.

## Data Availability

Data from the consortium will be deposited in public databases, and also made available online through the SCGE Toolkit (https://scge.mcw.edu/toolkit), as described in the SCGE Data Sharing Policy (available at https://scge.mcw.edu/policies/; periodically updated and amended by SCGE Consortium members).
