# Supplementary material for: The NIH Somatic Cell Genome Editing program
Source: Nature. 2021 Apr 7;592(7853):195–204. doi: 10.1038/s41586-021-03191-1 (PMC8026397; doi:10.1038/s41586-021-03191-1)
Supplement: Supplementary file 1 — Grouped membership list of the five SCGE initiatives. [file 41586_2021_3191_MOESM1_ESM.pdf]

nature portfolio
https://doi.org/10.1038/s41586-021-03191-1

Supplementary information

The NIH Somatic Cell Genome Editing program

In the format provided by the authors and unedited

Supplementary note

In addition to the authors listed in the main paper, the following consortium members are participants in the SCGE program:

Animal Reporter and Testing Center Initiative

Nicholas R. Anderson[1], Larry Bechtel[2], David Bergstrom[2], Benjamin Bimber[3], Chandra Boosani[4], Benjamin Burwitz[3], Tiffany Davis[2], Jeffery Duryea[2], Chris Fahrenkrug[5], Jonathan Green[4], Carrie Hanna[3], Seth Hannigan[2], Dennis J. Hartigan-O'Connor[6], Caleb Heffner[2], Raju Ilangovan[4], Dae Young Kim[7], Mark Krebs[2], Ralf Kühn[8], Denise Lanza[9], Eric Leffeler[4], Alexa Martinez[10], Matthew McKay[2], Branden Moriarity[11], Theresa Musket[4], Nadia Rosenthal[2], Timothy J. Safranski[4], Robert Schnabel[4], Susan Schommer[4], John Seavitt[9], Kathy Snow[2], Lukkana Suksanpaisan[12], Basile Tarchini[2], Christopher Walkey[10], Dennis Webster[5], Jonathan Wilde[13], Michael Wiles[2], Wolfgang Wurst[14]

Biological Effects Initiative

Amr Abdeen[15], Hye-Hyun Ahn[16], Alisha Birk[17], Elizabeth E. Capowski[18], Matthew Carter[17], Yong Cheng[19], Bruce Conklin[17,20], Nelly Cruz[21], Amritava Das[15], Lorena De Onate Monje[22], Micah Dombroe[15], Mo R. Ebrahimkhani[23], Olaia Fernandez Vila[17], Maria Fernandez Zepeda[24], Hongxia Fu[21], Kirstan Gimse[15], Stephen Gottschalk[25], Navin Gupta[26], Dennis J Hartigan-O'Connor[6], Louisa Helms[21], Jonathan Himmelfarb[27], Dirk Hockemeyer[22], Luke Judge[20], Serah Kang[17], Shalini Karthyk[28], John Kelly[29], Natalia G. Kolmakova[30], Giedre Krenciute[25], Katharina Kroll[31], Bonnie Lam[32], Kory Lavine[33], Cicera R. Lazzarotto[19], Andrea Lee[19], GaHyun Lee[19], Yichao Li[19], Jian Ma[34], Nick Malinin[19], Ronald Manlapaz[17], Nicholas Maragakis[35], Samantha Maragh[30], Krishna Maremanda[28], Reid Martin[25], Julie Mathieu[36], Oriane Matthys[17,37], Sunnie Grace McCalla[15], Tyler McCullough[38], Jean-Yves Metais[25], Il Minn[16], Kaivalya Molugu[15], Ray Monnat[39,40], Katherine Mueller[15], Mollie O'Brien[35], Jin Park[41], Bikash Pattnaik[42], Nicole K. Paulk[43], Viswesh Periswamy[15], Qian Qi[19], Sarah Rockwood[17], Hannele Ruohola-Baker[44], Kayvan Samimi[45], Shivani Saxena[15], David Schaffer[46], Ali Shakeri-Zadeh[16], Kelly Smith[47], Rupa Sridharan[15], Arens Taga[35], Tamara Traitteur[26], Mark Velasquez[32], Kayla Wolf[31], Ying Xia[29], Yang Yang[34], Wendy Yang[34], Tiffany Yee[19], Ruochi Zhang[34], Chengcheng Zhang[26]

Delivery Systems Initiative

Amr Abdeen[15], Safa Alfageeh[48], Michele Alves-Bezerra[10], Nadia Amrani[49], Samir Andrade Mendonça[50], Andrew Anzalone[48], Chris Baehr[51], Nathan Bamidele[49], Youmei Bao[52], Jote Bulcha[53], Doug Burrin[54], Ann T. Chen[55], Jiaxuan Chen[56], Zexiang Chen[49], Xiaoxia Cui[57], Agnieszka Czechowicz[58], Amritava Das[15], Saumya Das[59], Jessie R. Davis[48], Yanxiang Deng[55], Garth Devlin[60], Carla Dibb[58], Igor P. Dmitriev[50], Jordan L. Doman[48], Micah Dombroe[15], Wan Du[61], Dimas Echeverria Moreno[49], Nicholas G. Economos[62], Marie E. Egan[63], Sayo Eweje[56], Rong Fan[55], Xue Gao[64], Nicholas Gaston[49], Kirstan Gimse[15], Trevor Gonzalez[65], David Guay[66], Piotr Hadaczek[67], Stéphanie Hallée[66], Carolyn A. Haller[56], Patrick Havlik[60], Maomao He[68], Zhi Hong Lu[69], Mingqiang Huang[61], Tony P. Huang[48], Ayrea Hurley[10], Laxmi Jakkula[70], Allen Jiang[71], Zachary Kennedy[49], Luke W. Koblan[48], Elisa Konofagou[72], Sateesh Krishnamurthy[73], Katarina Kulhankova[73], Yeh-Hsing Lao[72], Sin Lee[64], Ang Li[64], Yamin Li[74], Yiran Li[61], Shun-

Qing Liang[49], Pengpeng Liu[75], Corena Loeb[61], Reka Lorincz[50], Kevin Luk[75], Alex Marson[76], Sunnie Grace McCalla[15], Rima Mendonsa[77], Jeanette Metzger[78], Shannon M. Miller[48], Kaivalya Molugu[15], Buhle Moyo[64], Katherine Mueller[15], Gregory A. Newby[48], Ogo Ojelabi[49], Bikash Pattnaik[42], Viswesh Periswamy[15], Alexandra S. Piotrowski-Daspit[55], Karthikeyan Ponnienselvan[75], Arun Prabhu Rameshbabu[61], Min Qiu[74], Elias Quijano[62], Maire Rayburn[58], Julia Rembetsy-Brown[49], Wei Ren[61], Lluis Samaranch[67], Kayvan Samimi[45], Shivani Saxena[15], Wendy Sheu[55], Rupa Sridharan[15], Kristyen Tomcik[79], Soumba Traore[73], Christopher Walkey[10], Dan Wang[53], Xiuxiu Wang[15], Yuyuan Wang[15], Yingjie Wu[61], Joshua Wythe[10], Ruosen Xie[15], Ying Xie[52], Lingling Xu[70], Fan Yang[70], Zhongfeng Ye[74], Han Zhang[49], Kevin Zhao[48], Yi Zhao[15], Wenliang Zhu[61]

Dissemination and Coordinating Center Initiative
Jeff De Pons[80], Arthur Derse[81], Aron M. Geurts[82], Michael Grzybowski[82], Matthew Hoffman[82], Anne E. Kwitek[82], Angela Lemke[82], Rebecca Schilling[82], Jennifer R. Smith[80], Ryan Spellecy[81], Aimee Steffenhagen[82], Anne Temple[82], Jyothi Thota[80], Monika Tutaj[80]

Genome Editors Initiative
Basem Al-Shayeb[83], Rodolphe Barrangou[84], Gabe Butterfield[38], Jessie R. Davis[48], Jordan L. Doman[48], Nicholas G. Economos[62], Jennifer Hamilton[22], Tony P. Huang[48], Nahid Iglesias[38], Bibekananda Kar[85], Luke W. Koblan[48], Shannon M. Miller[48], Kavini Nanayakkara[85], Gregory A. Newby[48], Patrick Pausch[77], Alexandra S. Piotrowski-Daspit[55], Elias Quijano[62], Tim Reddy[86], Ankit Sabharwal[85], Hyunjin Shim[83], Mark D. Wishman[85]

Affiliations
[1]Department of Public Health Sciences, Division of Health Informatics, School of Medicine, University of California, Davis, Davis, CA, 95616 [2]The Jackson Laboratory, Bar Harbor, ME 04609 USA; [3]Oregon National Primate Research Center, Oregon Health & Science University, Beaverton, OR 97006 USA; [4]College of Agriculture, Food & Natural Resources, University of Missouri, Columbia, Columbia, MO 65211 USA; [5]Recombinetics, Inc., Eagan, MN 55121 USA; [6]Department of Medical Microbiology and Immunology and California National Primate Research Center, University of California, Davis, Davis, CA 95616 USA; [7]College of Veterinary Medicine, University of Missouri, Columbia, Columbia, MO 65211 USA; [8]Max Delbrück Center for Molecular Medicine, 13125, Berlin, Germany; [9]Department of Molecular and Human Genetics, Baylor College of Medicine, Houston, TX 77030 USA; [10]Department of Molecular Physiology and Biophysics, Baylor College of Medicine, Houston, TX 77030 USA; [11]Department of Pediatrics, University of Minnesota Medical School, Minneapolis, MN 55454 USA; [12]Imanis Life Sciences, LLC, Rochester, MN 55901 USA; [13]McGovern Institute for Brain Research, Massachusetts Institute of Technology, Cambridge, MA 02139 USA; [14]Institute of Developmental Genetics, Helmholtz Zentrum München, D-85764 Neuherberg, Germany; [15]Wisconsin Institute for Discovery, University of Wisconsin-Madison, Madison, WI 53715 USA; [16]Department of Radiology, Johns Hopkins University School of Medicine, Baltimore, MD 21205 USA; [17]Gladstone Institute of Cardiovascular Disease, Gladstone Institutes, San Francisco, CA 94158 USA; [18]Waisman Center and McPherson Eye Research Institute, University of Wisconsin-Madison, Madison, WI 53715 USA; [19]Department of Hematology, St. Jude Children's Research Hospital, Memphis, TN 60656 USA; [20]Department of Medicine, University of California, San Francisco, San Francisco, CA 94158 USA; [21]Department of Medicine/Division of Nephrology, University of Washington, Seattle, WA 98195 USA;

[22]Department of Molecular and Cell Biology, University of California, Berkeley, Berkeley, CA 94720 USA; [23]Division of Experimental Pathology, Department of Pathology, member Pittsburgh Liver Research Center and McGowan Institute of Regenerative Medicine, University of Pittsburgh School of Medicine, Pittsburgh, PA 15261 USA; [24]Waisman Center, University of Wisconsin-Madison, Madison, WI 53715 USA; [25]Department of Bone Marrow Transplant and Cellular Therapy, St. Jude Children's Research Hospital, Memphis, TN 60656 USA; [26]Department of Medicine, Massachusetts General Hospital, Boston, MA 02114 USA; [27]Kidney Research Institute, University of Washington, Seattle, WA 98104 USA; [28]Department of Medicine, Brigham and Women's Hospital, Boston, MA 02116 USA; [29]Robarts Research Institute, Western University, London, ON N6A 5K8, Canada; [30]NIST, Gaithersburg, MD 20899 USA; [31]Wyss Institute for Biologically Inspired Engineering, Harvard University, Cambridge, MA 02138 USA; [32]Department of Bioengineering, University of California, Berkeley, Berkeley, CA 94720 USA; [33]John T. Milliken Department of Internal Medicine, Cardiovascular Division Washington University in St. Louis, St. Louis, MO 63110 USA; [34]Computation Biology Department, Carnegie-Mellon University, Pittsburgh, PA 15213 USA; [35]Department of Neurology, Johns Hopkins University School of Medicine, Baltimore, MD 21205 USA; [36]Department of Comparative Medicine, University of Washington, Seattle, WA 98195 USA; [37]Graduate Program in Bioengineering, University of California Berkeley/University of California, San Francisco, San Francisco, CA 94158 USA; [38]Department of Biomedical Engineering, Duke University, Durham, NC 27708 USA; [39]Department of Laboratory Medicine and Pathology, University of Washington, Seattle, WA 98195 USA; [40]Department of Genome Sciences, University of Washington, Seattle, WA 98195 USA; [41]Center for Personalized Diagnostics, Biodesign Institute, Arizona State University, Tempe, AZ 85281 USA; [42]Department of Pediatrics, University of Wisconsin-Madison, Madison, WI 53715 USA; [43]Department of Biochemistry and Biophysics, University of California, San Francisco, San Francisco, CA 94158 USA; [44]Department of Biochemistry, University of Washington, Seattle, WA 98195 USA; [45]Morgridge Institute for Research, Madison, WI 53715 USA; [46]Departments of Chemical and Biomolecular Engineering, Bioengineering, and Molecular and Cellular Biology, University of California, Berkeley, Berkeley, CA 94720 USA; [47]Department of Pathology, University of Washington, Seattle, WA 98195 USA; [48]Merkin Institute of Transformative Technologies in Healthcare, Broad Institute, Cambridge, MA 02142 USA; [49]RNA Therapeutics Institute, University of Massachusetts Medical School, Worcester, Worcester, MA 01605 USA; [50]Department of Radiation Oncology, Washington University in St. Louis, St. Louis, MO 63110 USA;[51]Department of Biomedical Engineering, University of California, Davis, Davis, CA 95616 USA; [52]Department of Neurosurgery, Yale University, New Haven, CT 06522 USA; [53]Horae Gene Therapy Center, University of Massachusetts Medical School, Worcester, MA 01605 USA; [54]Department of Pediatrics, Baylor College of Medicine, Houston, TX 77005 USA; [55]Department of Biomedical Engineering, Yale University, New Haven, CT 06520 USA; [56]Department of Surgery, Beth Israel Deaconess Medical Center, Boston, MA 02215 USA; [57]Genome Engineering & iPSC Center, Department of Genetics, Washington University in St. Louis, St. Louis, MO 63110 USA; [58]Department of Pediatrics, Stanford University, Stanford, CA 94304 USA; [59]Department of Cardiology, Massachusetts General Hospital, Boston, MA 02214 USA; [60]Department of Surgery, Duke University, Durham, NC 27707 USA; [61]Otolaryngology-Head and Neck Surgery, Massachusetts Eye & Ear Infirmary, Boston, MA 02114 USA; [62]Department of Genetics, Yale School of Medicine, New Haven, CT 06510 USA; [63]Department of Pediatrics, Yale School of Medicine, New Haven, CT 06510 USA; [64]Department of Bioengineering, Rice University, Houston, TX 77005 USA; [65]Department of Molecular Genetics and Microbiology, Duke University, Durham, NC 27708 USA; [66]Department of Research,

Feldan Therapeutics, Québec, QC G1P 4S6, Canada; [67]Department of Neurological Surgery, Ohio State University, Columbus, OH 43210 USA; [68]Department of Bioengineering, University of California, Berkeley, Berkeley, CA 94720 USA; [69]Department of Radiation Oncology, Washington University in St. Louis, St. Louis, MO 63110 USA; [70]Department of Pulmonary Immunology, University of Texas Health Sciences Center at Tyler, Tyler, TX 75708 USA; [71]David H. Koch Institute for Integrative Cancer Research, Massachusetts Institute of Technology, Cambridge, MA 02139 USA; [72]Department of Biomedical Engineering, Columbia University Health Sciences, New York, NY 10032 USA; [73]Department of Pediatrics, University of Iowa, Iowa City, IA. 52242 USA; [74]Department of Biomedical Engineering, Tufts University, Medford, MA 02155 USA; [75]Department of Molecular, Cell and Cancer Biology, University of Massachusetts Medical School, Worcester, Worcester, MA 01605 USA; [76]Departments of Microbiology and Immunology, University of California, San Francisco, San Francisco, CA 94143 USA; [77]Innovative Genomics Institute, University of California, Berkeley, Berkeley, CA 94720 USA; [78]Wisconsin National Primate Research Center, University of Wisconsin-Madison, Madison, WI 53715; [79]Department of Nutrition/Center for Proteomics and Bioinformatics, Case Western Reserve University, Cleveland, OH, 44106 USA; [80]Department of Biomedical Engineering, Medical College of Wisconsin, Milwaukee, WI 53226, USA; [81]Center for Bioethics and Medical Humanities, Medical College of Wisconsin, Milwaukee, WI 53226 USA; [82]Department of Physiology, Medical College of Wisconsin, Milwaukee, WI 53226 USA; [83]Department of Plant and Microbial Biology, University of California, Berkeley, Berkeley, CA 94720 USA; [84]Department of Food, Bioprocessing and Nutrition Sciences, North Carolina State University, Raleigh, NC 27695 USA; [85]Department of Biochemistry and Molecular Biology, Mayo Clinic Rochester, Rochester, MN 55905 USA; [86]Department of Biostatistics and Bioinformatics, Duke University, Durham, NC 27708 USA
